# Perforating Ulcer of the Foot

**Published:** 1908-01

**Authors:** M. G. Campbell

**Affiliations:** Atlanta, Georgia


### PERFORATING ULCER OF THE FOOT.

By Dr. M. G. Campbell, Atlanta, Ga.

---

In discussing the subject,—Perforating Ulcer of the Foot,—I desire to exhibit a patient that I treated some months ago for this complaint.

The patient treated was Mr. J.B.E., age 41, male, 6 feet 5inches high, weighing now 234, dairyman by trade.

Family history: It may be interesting to say that this man's father is still living, being in his 71st year, and enjoying good health. His mother died when 61 years old, after a lingering illness of bowel trouble, lasting two years.

There were ten children in his father's family, six living and in good health. One son died of a supposed attack of acute tuberculosis; one from an accident that injured his spine; another from la grippe; the fourth died after a two days illness following a hard days work of splitting rails.

Previous History: Our subject grew into a large, stout man, weighing at one time two hundred and eighty pounds. When he was about twenty years old, he received an injury to his back by straining in lifting a heavy log. He has suffered several times with attacks of lumbago, being in bed during two attacks. When a boy in the field at work he tells me he would get very thirsty and would drink large quantities of water, which did not satisfy his thirst but a very short time. His thirst has always been great and he has always drank a great deal of water, and consequently passed large quantities of urine. His hands and feet usually are dry and harsh and the skin is easily fissured. There are many callous places on the bottom of his feet, and he suffered a great deal from corns on his toes. One of these came on the plantar surface of the big toe on the left foot ten or fifteen years ago. It was persistent. In 1902, a tack in his shoe burrowed into this corn, causing it to become inflamed. It was infected and an abscess formed in December, 1902. Pus was discharged and shortly thereafter a watery fluid ran from an opening which persisted in the center of the callous spot. For three years following, it rose every two or three months or whenever undue pressure was applied to the toes, as that caused by wearing a new shoe, or doing very much walking.

This patient came to me about the 1st of August 1905 complaining of this toe. I found it swollen and inflamed. It was about one and one half times it's natural size. On the dorsal surface was an induration which was very tender like an abscess forming. The nail was hypertrophied and so diseased that it was nearly ready to fall off. On the plantar surface was a callous spot about double the size of a pea. In the center of this spot was an opening from which oozed a serous fluid. I ran a small probe into it and found that the instrument passed through the interphalangeal joint and lodged just under the skin on the dorsal surface. This was where the abscess had been and which was trying now to renew itself. Here, on the dorsal surface, was an old scar where an opening of a former abscess had healed. The callous growth had extended along the walls of the sinus, so that the probe gave very little pain when introduced and there was no bleeding when I removed the instrument. The bare bone could be felt with the probe. He suffered when he walked and it was very disagreeable on account of a wet sock all the time from the constant oozing from the joint and disagreeable odor arising therefrom. He was at a great deal of inconvenience in delivering his milk, as he could scarcely walk.

Treatment: I treated the toe with applications of ichthyol and antiphlogistine for several days, which reduced the swelling some and relieved his pain. I then cut the callous part away and enlarged the opening for better drainage. I used, also, carbolic acid and iodine in the sinus. I then curetted it as best I could under local anaesthesia. I suggested a general anaesthetic so as to be able to cut the sinus out and reset the ends of the bone to try for a better result. He could not spare the time from his business on account of his wife expecting to be confined in October. After that, he came back saying he was ready for me to do the operation. I treated him for two months with palliative measures, opening up the sinus on dorsal surface of the foot, as well as enlarging the opening on the plantar surface for thorough drainage. There was no improvement from what I did. Dr. A.H. Lindome was called in to consult with me about Oct. 10th 1907. We agreed to amputate the toe and set the time for the operation. Preparatory to the operation, I had Mr. E. to bring me specimens of his urine for examination. To my amazement, I found it loaded with sugar. My recollection is that the specific gravity was 1040, color pale, sweetish odor, no albumen, sugar in abundance. One or two drops of the urine produced a quick precipitate in Fehling's solution. I reported the findings to Dr. Lindome and we agreed that a general anaesthetic was out of the question. We feared diabetic coma and no local union.

I was in a quandary. I had treated him practically for two months without a perceptible benefit and I dared not resort to amputation. I had a static electrical machine in my office and some form of electricity suggested itself to me. I had read of some benefit from the High Frequency current in diabetes and local troubles where there was an improper nerve supply, so I decided on this mode. I gave him the first treatment on October 12th 1905. His condition at that time was about this: His weight, 220 lbs., urine, Sp. Gravity 1040, color very light, odor sweetish, no albumen, sugar very much loaded; quantity 5½ to 10 quarts in 24 hours. Skin was dry and harsh, fissures in both hands, large callosities on soles of both feet. All of his nails diseased more or less. The big toe on left foot was nearly one and one-half times its normal size, induration still on the dorsal surface. The nail thickened and necrotic, nearly ready to fall off. The sinus through the interphalangeal joint admitting a small slate pencil or a small high-frequency tube for treatment of the scar. You could see daylight through this opening, if the toe was held up to the light. I gave him sittings of fifteen to twenty minutes daily from Oct 12th to 28th 1905. Then he received treatment every other day until December 22nd 1905. In addition to this, he received for about two weeks, a pill of strychnine, iron, arsenic lithium, benzoate and quassin three times a day.

Result: I claim that the perforating ulcer of the foot was cured by the application of the High Frequency current. After a weeks treatment, the tissues began to put on a more healthy appearance and finally the induration was softened and absorbed. The toe nail came out healthy and smoothe. The sinus closed up gradually and the corn on the plantar surface gradually diminished. The joint was healed and the function restored so that he could use it as well as ever. You will see that the big toe is about one half shorter in proportion, than the next toe. You will also notice that the nail on this toe is not as smooth as described. He tells me that a cow stepped on it and it came out rough and somewhat atrophied at the end. He suffered in March 1906 from a slight relapse, but he came back to me and received four or five treatments. This started the process of repair again, which resulted in complete healing. He has since remained well.

I mention here, incidentally,that I treated the enlarged metatarso-phalangeal joint of this big toe a few times with a flat High Frequency Tube with a great deal of improvement.

We find him still with Diabetes Mellitus in as bad form as when he first came for treatment over two years ago.

I can not say that this was benefitted at all by the treatment. I have tried different lines of treatment in a limited way for this, but with no diminution of sugar in the urine. The treatment instituted relieved the local condition, but without removing the cause. His general condition was, however, improved to some extent.

I will say that to my mind there is no doubt that the condition of the toe was a true perforating ulcer of the foot as a result of Diabetis Mellitus.

Stelwagon, in his text-book on the skin, page 596, defines perforating ulcer of the foot as a trophoneurotic disease, beginning primarily as a degenerative, circumscribed, more or less calloused formation, and developing into an indolent, and usually painless, sinus, leading down through the deep tissues to the bóne.

Crocker says it is more correctly a sinus than an ulcer and often begins by a suppuration under a corn, burrowing into the soft tissues and when the horny covering is thrown off, a sinus is exposed leading down to the bare bone. Sometimes the process is more acute, and a slough is rapidly formed, but the result is the same, which is that the epidermis around the ulcer becomes much thickened and forms a thick, horny collar around the sinus, which is indolent, at times, painless, but occasionally hyperesthesia occurs, with a tendency to abundant and fetid perspiration of the affected foot.

Jackson speaks of the trouble as a process that slowly begins as a proliferation of the epidermis like a corn under which suppuration takes place and an ulcer is left.

Gross, in his surgery, page 1043, 1882 edition, says perforating ulcer begins as a callosity on the sole of the foot at the root of one of the matacarpal bones and gradually terminates in an undermined, ill looking ulcer with a dark, foul bottom and a peculiarly offensive sero-purulent discharge. It is surrounded with a wall of thickened epidermis and its edges are so steep as to give the appearance as if it had been made with a punch. It erodes the ligaments and periosteum and finally penetrates the contiguous joints followed by softening and caries of the bones.

The case in question, to my mind, is very accurately described by these writers in the above and in what they further say on the subject. Stelwagon, Crocker and Jackson all agree that the trouble may occur from diseases in which the nerve supply to the foot is deficient. It is a trophoneurosis and is dependent upon impairment or degeneration of the peripheral nerves. It occurs as a result of locomotor ataxia, anaesthetic leprosy, syphilis, peripheral neuritis or diabetes, etc.

In our case, we see that our patient is suffering even now from improper nerve supply or loss of tissue resisting power, as shown by his dry, cracked hands and callous patches on his feet.

The diagnosis is quite certain; simple suppurating corn or tuberculous ulcer are mentioned as the only confusing subjects. We find no history of tuberculosis. He does show diabetes with a damaged nerve supply of all of the extremities even now, as we have said in fissures and dryness of the hands and epidermal thickening and fissures of the feet. It commenced as a simple corn, but the other element of damage to nerve supply made it the perforating ulcer.

The treatment ordinarily recommended is about what I pursued in the beginning. Such as curetting, excission, stimulating applications and the like. Every writer that I have investigated says the treatment is unsatisfactory, even amputation being doubtful. Stelwagon says Treve's treatment is the most successful though when the bone is affected, that is doubtful. This consists in poulticing with linseed meal and shaving the callous part from day to day until this is removed. This requires ten to fourteen days. Then apply a paste composed of salicylic acid and glycerin in the consistency of cream with the addition of ten grains of carbolic acid.

In this case, the bone was diseased as shown by the probe. I must say that the satisfactory result in healing this condition by the use of the High Frequency current from an eight plate statis machine was indeed a great surprise to me and has been exceedingly interesting.

Our patient will testify that he has had great relief from this distressing malady for the past two years.

